# Detection of *Salmonella-*specific antibody in swine oral fluids

**DOI:** 10.1186/s40813-019-0136-7

**Published:** 2019-12-16

**Authors:** Briony M. Atkinson, Bradley L. Bearson, Crystal L. Loving, Jeffrey J. Zimmerman, Jalusa D. Kich, Shawn M. D. Bearson

**Affiliations:** 1 Food Safety and Enteric Pathogens Research Unit, USDA, ARS, National Animal Disease Center, 1920 Dayton Ave, Room 1403, Ames, IA 50010 United States; 2 Agroecosystems Management Research Unit, USDA, ARS, National Laboratory for Agriculture and the Environment, Ames, IA United States; 3 College of Veterinary Medicine, Iowa State University, Ames, IA United States; 4 Embrapa Swine and Poultry, Concórdia, SC Brazil

**Keywords:** *Salmonella*, Swine, Oral fluids, Antibody detection

## Abstract

*Salmonella* is a leading cause of bacterial foodborne-related illness and pork products are a food-associated source. With > 50% of U.S. swine herds testing positive for *Salmonella*, asymptomatic carrier pigs that shed *Salmonella* in their feces are a food safety and environmental contamination issue. Herd level surveillance of *Salmonella* shedding status is useful, but collection of feces and culture methods for *Salmonella* detection are laborious and time-consuming. Surveillance for *Salmonella*-exposure through detection of *Salmonella*-specific serum antibody is a reliable method, but presents labor and animal-welfare issues. Oral fluids are a reliable, antemortem sample with proven utility for surveillance in the swine industry. We tested oral fluid samples as a potential non-invasive, repeatable sample type for the presence of *Salmonella*-specific antibodies. An indirect enzyme-linked immunosorbent assay (ELISA) detected anti-*Salmonella* IgG, IgM, and predominantly IgA in oral fluids from *Salmonella enterica* serovar Typhimurium-exposed pigs. Furthermore, with minor modifications, a commercial ELISA-based kit also detected *Salmonella*-specific antibodies in oral fluids. Collectively, oral fluids may serve as a prospective surveillance tool for herd level monitoring of *Salmonella* exposure.

## Background

*Salmonella* is a common source of bacterial foodborne-related illness, with an estimated one million U.S. cases annually [1]. *Salmonella* was the causative agent of 762 foodborne outbreaks over the last 20 years, and pork products were the third highest associated food source with 10.8% attribution [2].

A 2006 study indicated that > 50% of swine production sites tested positive for *Salmonella* [3]. Identifying *Salmonella*-exposed herds is challenging because pigs are typically asymptomatic. Currently, surveillance for *Salmonella* on the farm is performed by bacteriological recovery of the organism in fecal samples or inferred through serological detection of *Salmonella-*specific antibodies. The swine industry could benefit from a *Salmonella* assay utilizing an antemortem sample with minimal animal stress and reduced labor for collection.

Oral fluids are commonly used to survey for infectious agents or antibody to specific organisms, and have improved whole herd surveillance programs [4–8]. To build on the utility of oral fluids for *Salmonella*-exposure surveillance, we used an immunoassay to detect *Salmonella*-specific antibody with oral fluids collected from pigs challenged with *Salmonella enterica* serovar Typhimurium (*S*. Typhimurium); the assay detected anti-*Salmonella* immunoglobulins (Ig) G, IgM, and predominantly IgA in oral fluids. Moreover, slight alterations to a commercial *Salmonella* antibody test for swine serum and meat juice samples detected anti-*Salmonella* immunoglobulins in oral fluids. Thus, detection of *Salmonella*-specific antibodies in oral fluids could function as a repeatable sample during the production cycle to provide not only timely surveillance information on *Salmonella* exposure and herd immunity, but also evaluate the effectiveness of disease intervention strategies against *Salmonella*.

## Material and methods

### Swine sample collection

Experiment 1. Eight-week-old cross-bred pigs (*n* = 3) testing negative for *Salmonella* in feces and for *Salmonella*-specific antibody in serum were housed in ABSL-2 isolation at the National Animal Disease Center (NADC), Ames, IA. Pigs were intranasally inoculated with 1 × 10^10^ nalidixic acid resistant *S.* Typhimurium strain UK-1 (SB377) [9]. Fecal samples were collected at 0, 2, and 14 days post-inoculation (dpi) for quantitative and qualitative *Salmonella* culture analyses, as previously described [10]. Samples were collected prior to challenge and at 34, 37, 43, 49, and 55 dpi for oral fluids and 34, 37, 49, and 56 dpi for serum. Oral fluids were collected by hanging cotton ropes (1/2″, Web Rigging Supply, Lake Barrington, IL) for ~ 30 min in the isolation room for the pigs to chew. The wet end of the rope was collected in a re-sealable plastic bag, and the bag was passed through a wringer (Dyna-jet BL-44, Overland Park, KS). Oral fluids were centrifuged (800×g, 20 min), filtered (45um-pore-size), and stored at -20 °C until assayed [11]. Blood was collected by venipuncture into 8.5 ml BD vacutainer serum separation tubes (BD, Franklin Lakes, NJ), centrifuged (1500×g, 10 min), and serum stored at -80 °C [9].

Experiment 2. Eight-week-old cross-bred pigs (*n* = 14) were housed in ABSL2 isolation and intranasally inoculated with 1 × 10^9^ SB377. Feces, serum, and oral fluids were collected and processed prior to inoculation (D0 or D-5 (5 days prior to inoculation)) and 15 dpi (D15) as described above.

### Heat-inactivated *Salmonella*

SB377 statically-grown (37 °C overnight) in LB broth (Invitrogen, Carlsbad, CA) was pelleted, washed, and resuspended in phosphate buffered saline (PBS). Bacteria were incubated at 65 °C for 45 min, aliquoted, and stored at -20 °C to serve as antigen in assays described below.

### Indirect enzyme-linked immunosorbent assay for *Salmonella*-specific antibody detection

NUNC Immuno Maxisorp Flat bottom 96-well plates (Thermofisher, Wilmington, DE) were coated with 0.2 ml heat-inactivated SB377 at 1 μg/ml and incubated at 4 °C for 18 h in a humid chamber. Following 3 washes (PBS), blocking buffer (1% bovine serum albumin (BSA) in PBS; B-PBS) was added (1 h, 23-25 °C). Oral fluids were diluted 1:2 with B-PBS. Serum was diluted 1:800 with B-PBS. Following 1 h incubation (23-25 °C), wells were washed 3X with 0.01% Tween 20 in B-PBS (B-PBS-T). Horseradish-Peroxidase conjugated antibody specific to swine IgA, IgM (1 mg/ml; Bethyl laboratories, Montgomery, TX) or IgG (0.5 mg/ml; Kirkegaard and Perry, Gaithersburg, MD) were diluted 1:20,000 and 1:80,000 in B-PBS-T for use in the oral fluid and serum ELISAs, respectively. Following 1 h incubation with secondary antibody (23-25 °C), plates were washed 3X with PBS, and 3,3′,5,5′-tetramethylbenzidine (TMB) stabilized chromogen (Thermofisher, Wilmington, DE) added for 30 min (23-25 °C, dark). Reactions were stopped with 1 N Sulfuric Acid (Honeywell, Charlotte, NC), and optical density (OD) was read at 450 nm on a BioTek synergy/HT microplate reader (BioTek, Winooski, VT) using GEN5 version 2.05 software. Results were reported as the average OD of duplicates of each sample. Limit of Detection (LOD) was calculated by determining the average OD and the standard deviation (SD) of the pre-challenge samples, multiplying the SD by three, and adding that number to the average OD of the pre-challenge samples. Data was analyzed using GraphPad Prism 7 software (GraphPad software Inc., La Jolla, CA).

### IDEXX HerdChek swine *Salmonella* antibody assay

In serum, porcine antibodies to *Salmonella* lipopolysaccharide (LPS) were measured as previously described using the IDEXX HerdChek Swine *Salmonella* Test Kit (IDEXX Europe B.V., Hoofddorp, Netherlands) [12]. To test oral fluids with the IDEXX kit, assay instructions were amended as follows: oral fluid was diluted 1:2 with sample diluent and incubated overnight (23-25 °C) in a humid chamber. Kit-supplied secondary antibody conjugate was incubated for 45 min (23-25 °C); assay development followed manufacturer’s recommendations. Reactions were measured at OD_650_ and converted to sample-to-positive (S/P) ratios. Ratios ≥0.25 were considered positive, while < 0.25 were considered negative. Samples provided with the kit as positive and negative controls were used to calculate S/*P* values for serum and oral fluid samples. In addition, oral fluids from experiment 1 was used as the negative (0 dpi) and positive (55 dpi) controls to calculate the S/P ratios for oral fluids from experiment 2, and vice versa (using 15 dpi from experiment 2 as positive control).

## Results & discussion

To determine if *Salmonella*-specific antibodies could be detected in swine oral fluids, a proof-of-concept study was performed. Pigs were challenged with *S.* Typhimurium at 10^10^ CFU (experiment 1) or 10^9^ CFU (experiment 2), and at 2 dpi, pigs shed an average of 5.4 X 10^5^ (+/− 3.1 × 10^5^; standard error of the mean (SEM)) CFU/g feces (experiment 1) and 6.1 X 10^5^ (+/− 2.6 × 10^5^) CFU/g feces (experiment 2). By 14–15 dpi, *Salmonella* fecal shedding averaged 1.5 X 10^3^ (+/− 1.3 × 10^3^) CFU/g feces (experiment 1) and 3.5 X 10^2^ (+/− 1.3 × 10^2^) CFU/g feces (experiment 2). Using an isotype-specific, in-house ELISA for IgA, IgG, and IgM, *Salmonella*-specific circulating antibodies were detected in the sera of both groups of pigs in response to inoculation with *Salmonella* SB377 (Additional file 1: Figure S1).

**Fig. 1:**
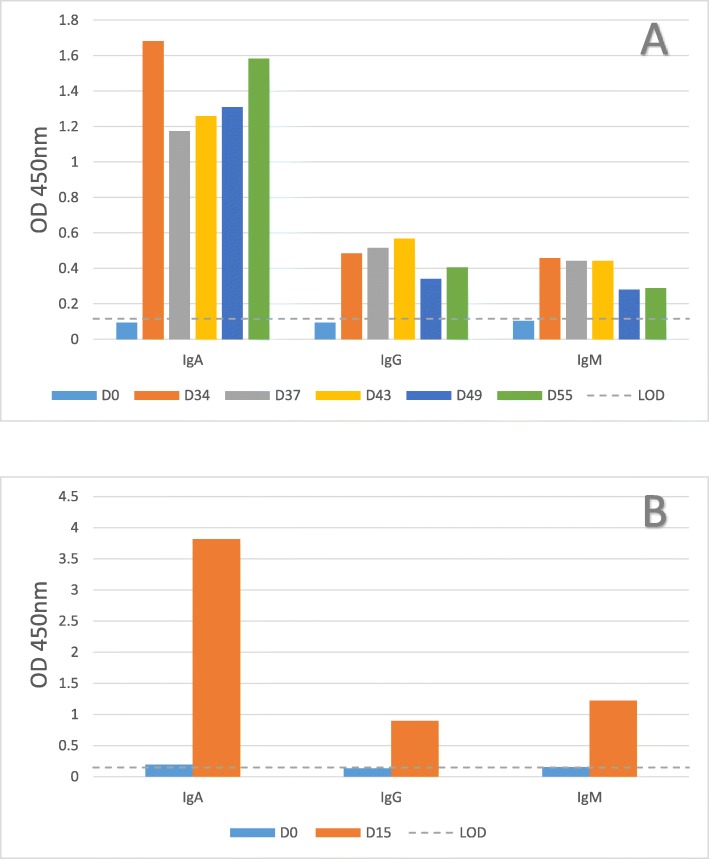
Detection of anti-*Salmonella* immunoglobulins in swine oral fluids. Oral fluid samples were collected from *S*. Typhimurium UK-1 challenged pigs (SB377) at the indicated day (D) relative to inoculation. Samples were evaluated for *Salmonella* SB377-specific IgA, IgG, and IgM antibody in an in-house ELISA. Oral fluid samples are a herd level sample, therefore only a single data point is presented for each collection date. Results from experiment 1 (*n* = 3 pigs) are presented in panel **A** and from experiment 2 (*n* = 14 pigs) in panel **B**. The horizontal dotted line denotes the limit of detection (LOD) set by the pre-challenge oral fluid samples

To determine if oral fluids contained antibodies against the *S*. Typhimurium strain, oral fluids collected at 34–55 dpi (experiment 1) and 15 dpi (experiment 2) were tested using the ELISA for *Salmonella*-specific IgA, IgG, and IgM. All post-inoculation oral fluids measured above the limit of detection set by the pre-challenge oral fluid samples, especially *Salmonella* SB377-specific IgA (Figure 1); thus, *Salmonella*-specific antibodies were present in oral fluids following experimental *Salmonella* inoculation. Higher levels of the *Salmonella*-specific IgA isotype were observed in the oral fluids (Figure 1) compared to a higher abundance of the IgG and IgM isotypes typically measured in the sera samples (Additional file 1: Figure S1). Neither serum nor oral fluid antibodies cross-reacted to *Escherichia coli* serotype O43:H28 strain 123 in the ELISA assay as all post-inoculation samples measured below the limit of detection set by the pre-challenge oral fluid samples (OD_450nm_ = 0.15) and pre-challenge sera samples (OD_450nm_ = 0.21).

We assessed if the anti-*Salmonella* immunoglobulins in oral fluids could be detected in the IDEXX HerdChek Swine *Salmonella* Test Kit, an ELISA assay commonly used in Europe to monitor *Salmonella* exposure [13, 14]. Although serum tested positive for antibodies to *Salmonella* LPS using the IDEXX assay (Additional file 1: Table S1), the oral fluids tested negative for antibodies using the kit-supplied positive and negative controls (Table 1, panel A). We presumed the lack of oral fluid antibody detection using the IDEXX assay was due to the nature of the proprietary controls supplied in the kit, which is assumed to be serum comprised of *Salmonella*-specific IgG and IgM, but containing little IgA; IgA was the predominant *Salmonella* SB377-specific isotype in the experimental oral fluids (based on Fig. 1). Alternatively, less antibody may be present in oral fluids, making it difficult to use the kit-supplied controls to calculate the sample-to-positive ratios. Therefore, we used oral fluids collected from *Salmonella*-inoculated pigs (positive control) and prior to inoculation (negative control) to calculate the sample-to-positive control ratios. All oral fluids collected after inoculation tested positive in the modified IDEXX assay, and the pre-inoculation oral fluids were negative (Table 1, panel B). Thus, *Salmonella* SB377-specific antibodies were detected in oral fluids using the IDEXX assay once the kit supplied controls for S/P calculation were replaced with oral fluid controls. Similar modifications were made by Kittawornrat et al. [5] to detect immunoglobulins against porcine reproductive syndrome virus (PRRSV) in oral fluids. The authors modified specific aspects of the IDEXX ELISA assay, including diluting the kit-supplied controls in order to calibrate the reactivity of the assay to the lower concentration of antibody present in oral fluids relative to sera. Our use of oral fluids as the negative and positive controls in the IDEXX assay achieved an analogous outcome. Furthermore, to compare IDEXX HerdCheck swine Salmonella ELISA results obtained from meat juice samples to sera samples, Wilhelm et al. applied a regression equation to recalculate the percent optical density data for meat juice samples in order to avoid underestimation of seroprevalence due to lower OD% levels for meat juice samples [14]. For this commercial kit (or others) to be utilized to query swine oral fluid samples for *Salmonella*-specific antibodies, additional development is needed to optimize assay performance, including sensitivity and specificity validation. Altogether, the data suggested that commercially available IDEXX HerdChek Swine *Salmonella* Test Kit can identify *Salmonella*-specific immunoglobulins in pig oral fluids when using oral fluids to set S/P ratios. 
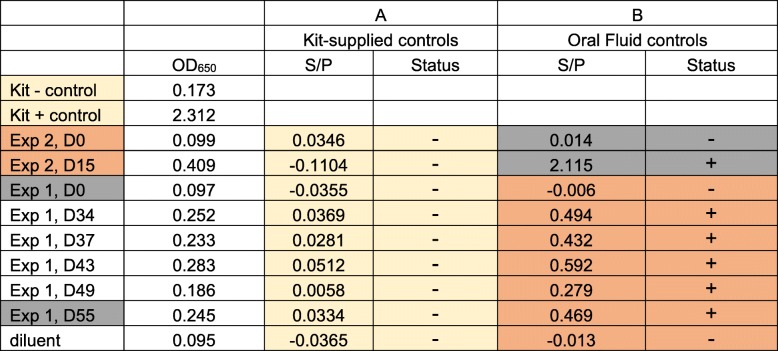

**Table 1:**
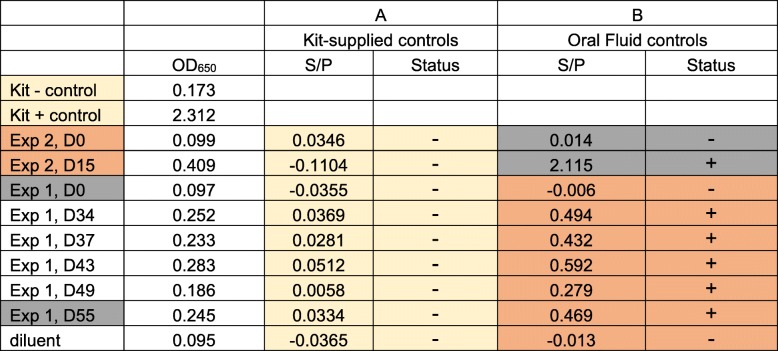
Anti-*Salmonella* immunoglobulins are detected in swine oral fluids with slight modifications to the IDEXX HerdChek Swine *Salmonella* Test Kit

Oral fluid samples from experiment 2 served as the negative oral fluid control (D0, prior to inoculation) and positive oral fluid control (D15, post-inoculation) for calculating sample-to-positive (S/P) ratios for experiment 1 (orange highlight). Oral fluid samples from experiment 1 served as the negative oral fluid control (D0, prior to inoculation) and positive oral fluid control (D55, post-inoculation) for calculating S/P ratios for experiment 2 (gray highlight). S/P ratios for the oral fluid samples highlighted in yellow were calculated using the kit-supplied negative control (− control) and positive control (+ control). S/P ratios were calculated using optical density (OD) measurements in the following formula:

S/P = (OD_sample_ – OD_NC_)/(OD_PC_ – OD_NC_)

Ratios ≥0.25 were considered positive while < 0.25 were considered negative

## Conclusion

Our proof-of-concept study indicated that immunoglobulins against *Salmonella* are detectable in oral fluids. An oral fluid-based assay as a surrogate for serum could serve as a surveillance tool to ascertain on-farm *Salmonella* exposure, improve swine health management decisions, and evaluate intervention strategies. Furthermore, determination of *Salmonella* exposure using oral fluids could allow proactive classification of *Salmonella* herd status while using a non-invasive, antemortem collection method.

## Supplementary information

**Additional file 1:** **Figure S1.** Detection of anti-*Salmonella* immunoglobulins in swine sera. Serum samples were collected from *S*. Typhimurium UK-1 challenged pigs (SB377) at the indicated day (D) relative to inoculation. Samples were evaluated for *Salmonella* SB377-specific IgA, IgG, and IgM antibody in an in-house ELISA. **Table S1.** A. Experiment 1 sera samples in IDEXX HerdChek Swine Salmonella ELISA. B. Experiment 2 sera samples in IDEXX HerdChek Swine Salmonella ELISA.

## Data Availability

Data requests should be directed to the corresponding author.

## Abbreviations

CFU: Colony forming units
dpi: days post-inoculation
ELISA: Enzyme-linked immunosorbent assay
IgA: Immunoglobulin A
IgG: Immunoglobulin G
IgM: Immunoglobulin M
LOD: Level of detection
OD: Optical density
S/P: Sample-to-positive
SEM: Standard error of the mean
