# Supplementary material for: Detection of Salmonella-specific antibody in swine oral fluids
Source: Porcine Health Manag. 2019 Dec 16;5:29. doi: 10.1186/s40813-019-0136-7 (PMC6915926; doi:10.1186/s40813-019-0136-7)
Supplement: Supplementary file 1 — Additional file 1: Figure S1. Detection of anti-Salmonella immunoglobulins in swine sera. Serum samples were collected from S. Typhimurium UK-1 challenged pigs (SB377) at the indicated day (D) relative to inoculation. Samples were evaluated for Salmonella SB377-specific IgA, IgG, and IgM antibody in an in-house ELISA. Table S1. A. Experiment 1 sera samples in IDEXX HerdChek Swine Salmonella ELISA. B. Experiment 2 sera samples in IDEXX HerdChek Swine Salmonella ELISA. [file 40813_2019_136_MOESM1_ESM.docx]

**Figure S1.** **Detection of anti-*Salmonella* immunoglobulins in swine sera**. Serum samples were collected from *S*. Typhimurium UK-1 challenged pigs (SB377) at the indicated day (D) relative to inoculation. Samples were evaluated for *Salmonella* SB377-specific IgA, IgG, and IgM antibody in an in-house ELISA.

| **Table S1A. Experiment 1 sera samples in IDEXX HerdChek Swine Salmonella ELISA** | | | | | | | | |
| --- | --- | --- | --- | --- | --- | --- | --- | --- |
|  |  | OD 650nm | | Average OD | S/P | Status |  |  |
|  | Kit - | 0.106 | 0.106 | 0.106 |  |  |  |  |
|  | Kit + | 0.942 | 0.904 | 0.923 |  |  |  |  |
| Pig 36 | D0 | 0.125 | 0.127 | 0.126 | 0.024 | - |  |  |
| Pig 36 | D37 | 0.856 | 1.082 | 0.969 | 1.056 | + |  |  |
| Pig 36 | D49 | 1.601 | 1.591 | 1.596 | 1.824 | + |  |  |
| Pig 36 | D55 | 1.454 | 1.350 | 1.402 | 1.586 | + |  |  |
| Pig 37 | D0 | 0.137 | 0.145 | 0.141 | 0.043 | - |  |  |
| Pig 37 | D37 | 1.640 | 1.432 | 1.536 | 1.750 | + |  |  |
| Pig 37 | D49 | 1.704 | 1.805 | 1.755 | 2.018 | + |  |  |
| Pig 37 | D55 | 1.700 | 1.708 | 1.704 | 1.956 | + |  |  |
| Pig 3899 | D0 | 0.509 | 0.566 | 0.538 | 0.528 | + |  |  |
| Pig 3899 | D37 | 1.812 | 1.680 | 1.746 | 2.007 | + |  |  |
| Pig 3899 | D49 | 2.051 | 2.070 | 2.061 | 2.392 | + |  |  |
| Pig 3899 | D55 | 2.392 | 2.330 | 2.361 | 2.760 | + |  |  |
|  | diluent | 0.090 | 0.092 | 0.091 | -0.018 | - |  |  |
|  |  |  |  |  |  |  |  |  |
|  |  |  |  |  |  |  |  |  |
| **Table S1B. Experiment 2 sera samples in IDEXX HerdChek Swine Salmonella ELISA** | | | | | | | | |
|  |  | OD 650nm | | Average OD | S/P | Status |  |  |
|  | Kit - | 0.075 | 0.072 | 0.074 |  |  |  |  |
|  | Kit + | 1.494 | 1.544 | 1.519 |  |  |  |  |
| Pig 801 | D0 | 0.07 | 0.069 | 0.070 | -0.003 | - |  |  |
| Pig 802 | D0 | 0.097 | 0.106 | 0.102 | 0.019 | - |  |  |
| Pig 803 | D0 | 0.072 | 0.075 | 0.074 | 0.000 | - |  |  |
| Pig 807 | D0 | 0.053 | 0.056 | 0.055 | -0.013 | - |  |  |
| Pig 810 | D0 | 0.08 | 0.076 | 0.078 | 0.003 | - |  |  |
| Pig 812 | D0 | 0.07 | 0.067 | 0.069 | -0.003 | - |  |  |
| Pig 813 | D0 | 0.068 | 0.064 | 0.066 | -0.005 | - |  |  |
| Pig 815 | D0 | 0.054 | 0.055 | 0.055 | -0.013 | - |  |  |
| Pig 816 | D0 | 0.126 | 0.113 | 0.120 | 0.032 | - |  |  |
| Pig 818 | D0 | 0.053 | 0.052 | 0.053 | -0.015 | - |  |  |
| Pig 819 | D0 | 0.05 | 0.053 | 0.052 | -0.015 | - |  |  |
| Pig 824 | D0 | 0.055 | 0.055 | 0.055 | -0.013 | - |  |  |
| Pig 825 | D0 | 0.053 | 0.051 | 0.052 | -0.015 | - |  |  |
| Pig 827 | D0 | 0.052 | 0.052 | 0.052 | -0.015 | - |  |  |
|  |  |  |  |  |  |  |  |  |
|  |  | OD 650nm | | Average OD | S/P | Status |  |  |
|  | Kit - | 0.116 | 0.122 | 0.119 |  |  |  |  |
|  | Kit + | 1.468 | 1.510 | 1.489 |  |  |  |  |
| Pig 801 | D15 | 0.824 | 0.741 | 0.783 | 0.484 | + |  |  |
| Pig 802 | D15 | 1.660 | 1.608 | 1.634 | 1.106 | + |  |  |
| Pig 803 | D15 | 1.795 | 1.784 | 1.790 | 1.219 | + |  |  |
| Pig 807 | D15 | 2.412 | 2.330 | 2.371 | 1.644 | + |  |  |
| Pig 810 | D15 | 2.734 | 2.654 | 2.694 | 1.880 | + |  |  |
| Pig 812 | D15 | 2.718 | 2.792 | 2.755 | 1.924 | + |  |  |
| Pig 813 | D15 | 2.672 | 2.616 | 2.644 | 1.843 | + |  |  |
| Pig 815 | D15 | 1.294 | 1.649 | 1.472 | 0.987 | + |  |  |
| Pig 816 | D15 | 0.727 | 2.226 | 1.477 | 0.991 | + |  |  |
| Pig 818 | D15 | 2.565 | 2.576 | 2.571 | 1.789 | + |  |  |
| Pig 819 | D15 | 1.399 | 1.231 | 1.315 | 0.873 | + |  |  |
| Pig 824 | D15 | 2.443 | 2.520 | 2.482 | 1.724 | + |  |  |
| Pig 825 | D15 | 1.951 | 1.634 | 1.793 | 1.222 | + |  |  |
| Pig 827 | D15 | 0.873 | 1.203 | 1.038 | 0.671 | + |  |  |
|  | diluent | 0.089 | 0.085 | 0.087 | -0.023 | - |  |  |
